# Capivasertib-Induced Diabetes Successfully Managed With Insulin-Independent Glucose-Lowering Agents: A Case Report

**DOI:** 10.7759/cureus.105623

**Published:** 2026-03-21

**Authors:** Ysutaka Takeda, Mariko Tsuyama, Yusuke Haba, Masafumi Inokuchi, Naoki Kumashiro

**Affiliations:** 1 Diabetology and Endocrinology, Kanazawa Medical University, Uchinada, JPN; 2 Breast Oncology, Kanazawa Medical University, Uchinada, JPN

**Keywords:** alpha-glucosidase inhibitor, breast cancer, capivasertib, diabetes mellitus, sodium-glucose cotransporter-2 (sglt-2) inhibitor

## Abstract

Capivasertib, an AKT (protein kinase B) inhibitor, in combination with fulvestrant, reduces the risk of progression in recurrent breast cancer; however, it frequently leads to hyperglycemia by disrupting the insulin signaling pathways. Insulin-based therapies are generally ineffective in this setting and may worsen cancer outcomes. Herein, we report a case of capivasertib-induced diabetes successfully managed with insulin-independent glucose-lowering agents while capivasertib therapy was continued. A 57-year-old female with a body mass index of 23.0 kg/m² developed hyperglycemia one month after initiating capivasertib, with a glycosylated hemoglobin (HbA1c) level of 7.3%. A 75-g oral glucose tolerance test (OGTT) confirmed the diagnosis of diabetes according to the American Diabetes Association diagnostic criteria, demonstrating a fasting glucose level of 173 mg/dL, a two-hour glucose level of 512 mg/dL, and marked hyperinsulinemia. The markedly elevated glucose levels observed during the OGTT likely reflect severe drug-induced hyperglycemia associated with AKT inhibition, rather than a typical OGTT response in untreated diabetes. Treatment with empagliflozin (10 mg/day, a sodium-glucose cotransporter-2 inhibitor) and voglibose (0.4 mg/day, an alpha-glucosidase inhibitor) improved the HbA1c level to 6.3% within two months. After five months, a repeat OGTT showed improvement, shifting from the level of diabetes to impaired glucose tolerance, with substantially reduced insulin levels. This case may represent one of the early reports describing the successful management of capivasertib-induced diabetes with insulin-independent glucose-lowering agents, suggesting a potential strategy for managing AKT inhibitor-induced hyperglycemia.

## Introduction

Capivasertib is an orally administered AKT (protein kinase B) inhibitor that targets all three AKT isoforms, demonstrates antitumor effects, and is used as a second-line treatment for breast cancer [1]. The CAPItello-291 study, a phase 3 trial involving Japanese patients with estrogen receptor-positive, HER2-negative, unresectable, or recurrent breast cancer who progressed after endocrine therapy with an aromatase inhibitor, demonstrated that combination therapy with capivasertib and fulvestrant (a selective estrogen receptor degrader) reduced the risk of disease progression or death by 50% compared to fulvestrant monotherapy [2]. However, because capivasertib inhibits insulin signaling via AKT, leading to reduced glucose uptake in peripheral tissues, increased hepatic glucose production, and marked insulin resistance, hyperglycemia is a known adverse effect. In fact, 16.3% of participants in the phase 3 trial developed hyperglycemia [2], and diabetic ketoacidosis (DKA) was reported as a serious adverse event. Rodriguez et al. recently described a case of capivasertib-induced DKA in a patient without a prior history of diabetes, with hyperglycemia showing serum glucose level of 1558 mg/dL refractory to high-dose insulin therapy, requiring three days of infusion therapy before transitioning to sliding scale mealtime insulin, and the case required over a week of intensive care [3]. These findings suggest that insulin or insulin-dependent glucose-lowering agents may be less effective in some cases of capivasertib-induced diabetes because profound insulin resistance induced by AKT inhibition can limit the glucose-lowering effect of exogenous insulin.

Here, we report a case of capivasertib-induced diabetes in a patient with recurrent breast cancer successfully managed with insulin-independent glucose-lowering agents.

This article was previously presented as a meeting abstract at the 99th Annual Meeting of the Japan Diabetes Society Chubu Branch on September 6, 2025.

## Case presentation

A 57-year-old postmenopausal female with estrogen receptor-positive, HER2-negative metastatic recurrence of breast cancer without a prior history of diabetes or obesity presented to our department with a glycosylated hemoglobin (HbA1c) level of 7.3% (56 mmol/mol) one month after beginning treatment with capivasertib (400 mg orally twice daily, four consecutive days on, three days off). She was 154.6 cm tall and weighed 55.0 kg, with a body mass index of 23.0 kg/m². Laboratory findings at initial presentation are summarized in Table 1.

**Table 1:** Laboratory data at initial presentation. AST: aspartate aminotransferase; ALT: alanine aminotransferase; G-GTP: gamma-glutamyl transferase; BUN: blood urea nitrogen; eGFR: estimated glomerular filtration rate; HbA1c: glycosylated hemoglobin.

Variables	Results	Reference range
Urine		
Protein	Negative	Negative
Glucose	3+	Negative
Ketone body	Negative	Negative
Complete blood count		
White blood cells (/µL)	4960	3300 - 8600
Neutrophils (%)	41.3	42.0 - 75.6
Lymphocytes (%)	34.5	17.4 - 48.2
Monocytes (%)	7.7	3.4 - 9.0
Eosinophils (%)	14.9	0.4 - 8.6
Basophils (%)	1.6	0.2 - 1.4
Red blood cells (×10^4^/µL)	466	435 - 555
Hemoglobin (g/dL)	13.9	13.7 - 16.8
Hematocrit (%)	41.4	40.7 - 50.1
Platelets (×10^4^/µL)	33.9	15.8 - 34.8
Biochemistry		
Total protein (g/dL)	7.1	6.6 - 8.1
Albumin (g/dL)	4.6	4.1 - 5.1
AST (U/L)	25	13 - 30
ALT (U/L)	32	10 - 42
G-GTP (U/L)	77	13 - 64
BUN (mg/dL)	9	8 - 20
Creatinine (mg/dL)	0.61	0.65 - 1.07
eGFR (mL/min/1.73 m^2^)	77.5	
Sodium (mmol/L)	143	138 - 145
Potassium (mmol/L)	3.8	3.6 - 4.8
Chloride (mmol/L)	106	101 - 108
Glucose metabolism		
Plasma glucose (mg/dL)	118	73 - 109
HbA1c (%)	7.3	4.9 - 6.0

**Figure 1:**
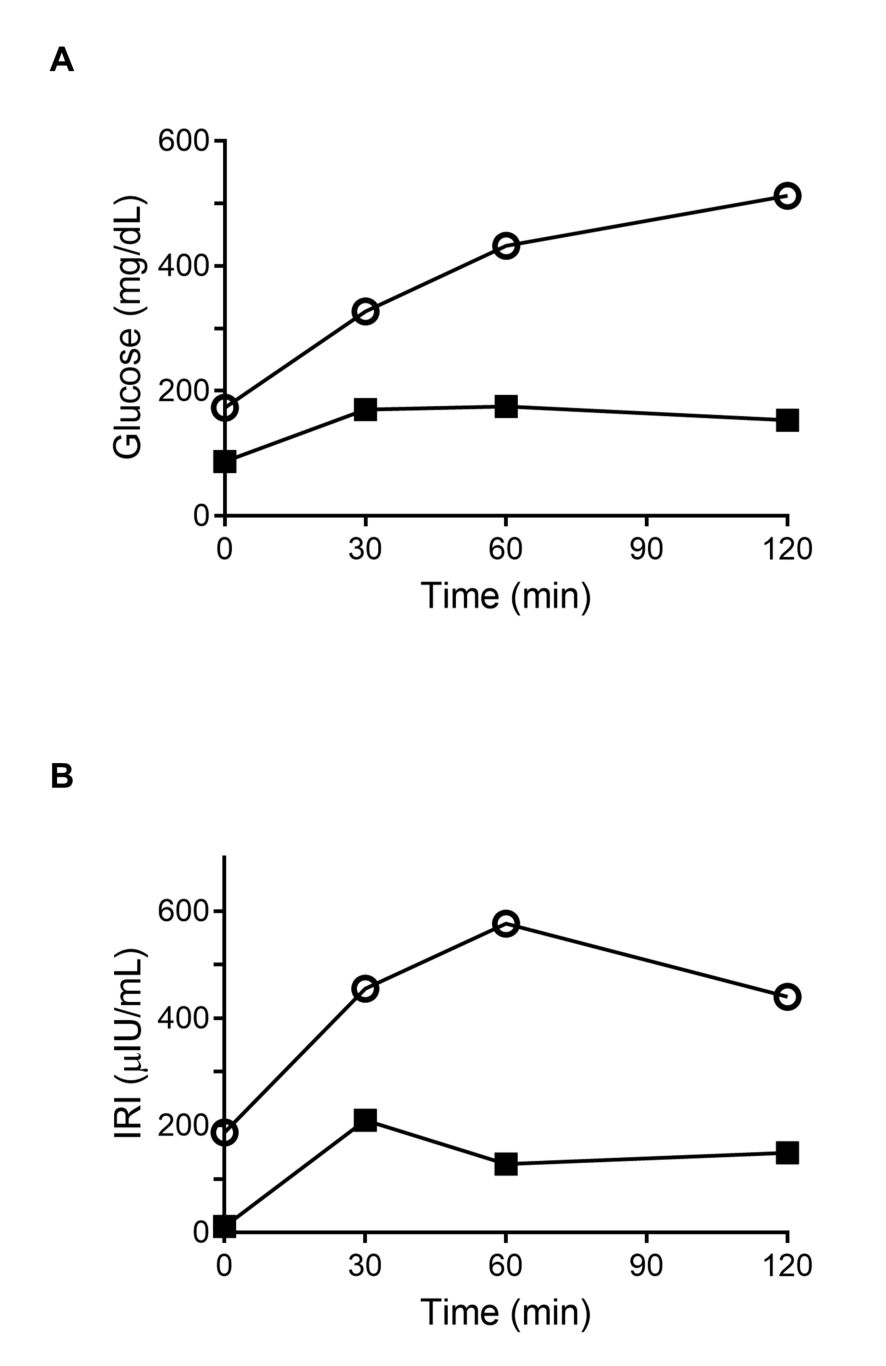
Results of the 75-g OGTT before and five months after treatment. A: Glucose levels during the OGTT. B: IRI levels during OGTT. Open circles: before treatment; closed squares: five months after treatment. OGTT: oral glucose tolerance test; IRI: immunoreactive insulin.

As shown in Figure 1A, she was diagnosed with diabetes based on the 75-g oral glucose tolerance test (OGTT) results, which revealed severe hyperglycemia, with fasting and two-hour plasma glucose levels of 173 and 512 mg/dL, respectively, indicating a potential hyperglycemic emergency, but the diagnostic criteria for hyperglycemic emergencies such as DKA or hyperosmolar hyperglycemic state were not met. The patient also exhibited marked hyperinsulinemia with a one-hour plasma insulin level of 576.4 μU/mL (Figure 1B).

Given that capivasertib inhibits all AKT isoforms, insulin therapy or insulin secretagogues may be less effective in some cases because of profound insulin resistance. Additionally, biguanides and thiazolidinediones were avoided because of theoretical concerns regarding potential enhancement of insulin signaling in the context of AKT inhibition. However, we acknowledge that metformin has been widely investigated for its potential anticancer effects in breast cancer and other malignancies. Therefore, the decision to avoid metformin in this patient reflects a cautious clinical judgment rather than an established contraindication. Thus, insulin-independent agents were selected as the preferred treatment option. While capivasertib treatment was continued at the initial dose, antidiabetic treatment was initiated with empagliflozin (10 mg/day, a sodium-glucose cotransporter-2 (SGLT2) inhibitor) and voglibose (0.4 mg/day, an alpha-glucosidase inhibitor), with the dose of voglibose later increased to 0.6 mg/day after two months. After two months, her HbA1c level improved to 6.3% (44 mmol/mol), and by five months, a repeat OGTT indicated an improvement, showing only impaired glucose tolerance with a marked reduction in hyperinsulinemia (Figure 1). Her body weight remained stable throughout the treatment. Because AKT inhibition may enhance lipolysis and ketogenesis, careful monitoring for ketosis is important when SGLT2 inhibitors are used. In this case, serum 3-hydroxybutyrate levels were largely unchanged (190 µmol/L at baseline and 202 µmol/L at five months post treatment; reference range = 0-85 μmol/L), and no clinical signs of ketosis were observed at any time.

## Discussion

The present case demonstrates a potential management strategy of capivasertib-induced diabetes using insulin-independent glucose-lowering agents. To date, no standard strategy exists for glycemic control in patients with breast cancer receiving AKT inhibitors, and our experience may provide valuable clinical guidance.

Capivasertib-induced AKT inhibition causes severe insulin resistance, resulting in hyperglycemia and potentially ketosis via enhanced lipolysis. Although treatment with SGLT2 inhibitors may exacerbate this risk, no ketosis was observed in our patient. Two potential mechanisms may explain these findings. First, insulin suppresses lipolysis via AKT-dependent and -independent pathways [4], with the independent pathway possibly preventing the development of ketosis. Second, selective insulin resistance, characterized by different insulin sensitivities between glucose and lipid metabolism, may also play a role [5,6].

As previously mentioned, insulin-independent glucose-lowering medications considerably reduced plasma insulin levels in our patient. However, the outcome could not be construed as evidence of improved insulin resistance because treatment with capivasertib, an AKT inhibitor, was continued, and the administration of SGLT2 and alpha-glucosidase inhibitors promoted glucose excretion without affecting insulin action. We propose that the marked reduction in insulin levels after glucose-lowering therapy in the present case was largely attributed to diminished glucose-responsive insulin secretion, resulting from decreased plasma glucose concentration via glucose excretion by SGLT2 and alpha-glucosidase inhibitors. Hyperinsulinemic-euglycemic clamp tests are necessary for the definitive evaluation of insulin sensitivity [7]; thus, it remains unclear whether insulin sensitivity improves. However, decreased plasma insulin concentration per se might be beneficial for preventing tumor growth [8], and no tumor progression was observed in our case, at least during the observation period. Thus, the treatment with insulin-independent glucose-lowering medications, such as SGLT2 and alpha-glucosidase inhibitors, may be a potential management strategy for capivasertib-induced diabetes in patients with recurrent breast cancer, which requires further investigation.

We successfully achieved glycemic control in a patient with breast cancer who developed capivasertib-induced diabetes; however, clinical challenges remain in the treatment of patients who develop hyperglycemic emergencies, including DKA or hyperglycemic hyperosmolar state. To date, three cases of hyperglycemic emergencies associated with capivasertib treatment have been reported [3,9,10]. Two of these patients recovered from critical illness [9,10], whereas the remaining patient did not show any improvement in her condition despite high-dose insulin therapy [3]. There is no doubt that insulin therapy should be used to treat hyperglycemic emergencies. However, at an earlier stage, before the development of DKA or hyperglycemic hyperosmolar state, the use of insulin-independent glucose-lowering agents may effectively and safely prevent hyperglycemic emergencies associated with capivasertib treatment.

## Conclusions

To the best of our knowledge, the present case first illustrates that early administration of insulin-independent glucose-lowering agents may represent a potential therapeutic strategy for managing AKT inhibitor-induced diabetes that warrants further investigation. Owing to its therapeutic potential as a second-line treatment for breast cancer, capivasertib is projected to gain wider utilization in clinical practice. We hope that our report will serve as a valuable resource for clinicians managing AKT inhibitor-induced diabetes.
